# Diaqua­sodium(I) perchlorate bis­[μ-2-(carboxyl­atomethyl­imino­meth­yl)phenolato]bis­[(3-methyl­pyridine)copper(II)]

**DOI:** 10.1107/S1600536808039561

**Published:** 2008-11-29

**Authors:** Xiangru Wen, Yongmin Liu, Zhengyi Li

**Affiliations:** a Xuzhou Medical College, Xuzhou 221004, People’s Republic of China

## Abstract

In the title compound, [Na(H_2_O)_2_]ClO_4_·[Cu_2_(C_9_H_7_NO_3_)_2_(C_6_H_7_N)_2_], the Cu^II^ atom is coordinated by one N atom and two O atoms from a tridentate *N*-salicylideneglycinate Schiff base dianion and one N atom from a 3-methyl­pyridine ligand. Longer Cu⋯O contacts [2.680 (2) Å] complete an approximate square-based pyramidal coordination geometry around Cu^II^, forming a dimeric complex across a centre of inversion. The dimeric complexes form stacks along the *a* axis, with Cu⋯O contacts of 3.332 (2) Å between them. The Na^+^ cations and perchlorate anions lie on twofold rotation axes between the stacks. The former are coordinated by two disordered water mol­ecules (each with half-occupancy), and form Na⋯O contacts of 3.698 (3) Å to the perchlorate anions and Na⋯π contacts to neighbouring salicylideneglycinate ligands [shortest Na⋯C = 3.516 (3) Å].

## Related literature

For related structures, see: Warda (1998*a* ▶,*b* ▶,*c* ▶,*d* ▶). For synthesis details, see: Ueki *et al.* (1967 ▶); Warda (1994 ▶).
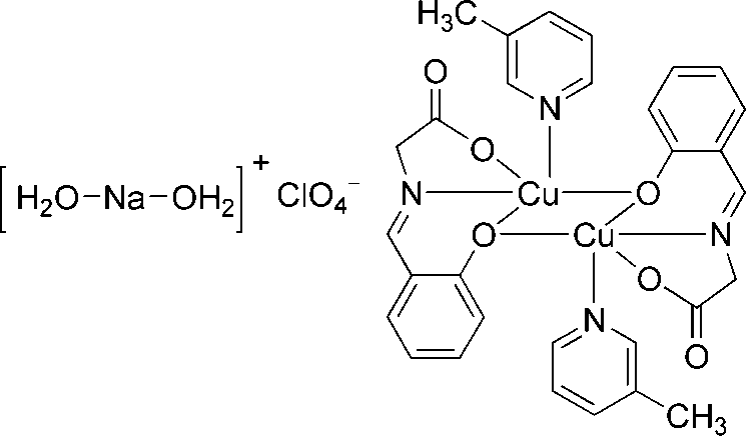


## Experimental

### 

#### Crystal data

[Na(H_2_O)_2_]ClO_4_·[Cu_2_(C_9_H_7_NO_3_)_2_(C_6_H_7_N)_2_]
*M* *_r_* = 826.12
Monoclinic, 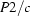

*a* = 7.4677 (5) Å
*b* = 13.2402 (9) Å
*c* = 19.3937 (12) Å
β = 109.165 (2)°
*V* = 1811.3 (2) Å^3^
*Z* = 2
Mo *K*α radiation
μ = 1.32 mm^−1^
*T* = 292 (2) K
0.30 × 0.26 × 0.24 mm

#### Data collection

Bruker SMART APEX CCD diffractometer
Absorption correction: multi-scan (*SADABS*; Bruker, 2000 ▶) *T*_min_ = 0.68, *T*_max_ = 0.73
18486 measured reflections
3558 independent reflections
2926 reflections with *I* > 2σ(*I*)
*R*_int_ = 0.047

#### Refinement

*R*[*F* ^2^ > 2σ(*F* ^2^)] = 0.045
*wR*(*F* ^2^) = 0.100
*S* = 1.13
3558 reflections
237 parameters
H-atom parameters constrained
Δρ_max_ = 0.29 e Å^−3^
Δρ_min_ = −0.59 e Å^−3^

### 

Data collection: *SMART* (Bruker, 2000 ▶); cell refinement: *SAINT* (Bruker, 2000 ▶); data reduction: *SAINT*; program(s) used to solve structure: *SHELXTL* (Sheldrick, 2008 ▶); program(s) used to refine structure: *SHELXTL*; molecular graphics: *SHELXTL*; software used to prepare material for publication: *SHELXTL*.

## Supplementary Material

Crystal structure: contains datablocks global, I. DOI: 10.1107/S1600536808039561/bi2318sup1.cif

Structure factors: contains datablocks I. DOI: 10.1107/S1600536808039561/bi2318Isup2.hkl

Additional supplementary materials:  crystallographic information; 3D view; checkCIF report

## Figures and Tables

**Fig. 1.**
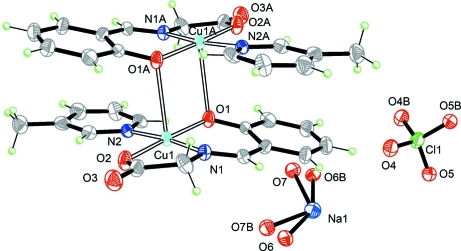
The structure of the title compound, showing 30% probability displacement ellipsoids for non-H atoms [symmetry codes: (A) 1 - x, 2 - y, 1 - z; (B) -x, y, -z + 1/2]. H atoms of the water molecules are omitted.

**Fig. 2.**
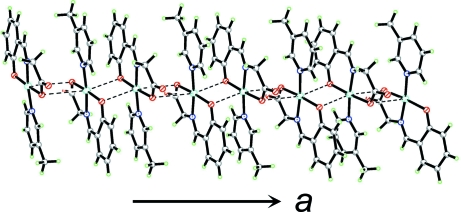
One-dimensional stack running along the a axis.

## supplementary crystallographic information

### Comment

Copper(II) complexes with tridentate Schiff-base dianions of the *N*-salicylideneaminoacidato type (TSB^2-^) have been reported recently (1998*a*–*d*). These complexes are pyridine adducts. In this paper, we present the structure of the related title compound.

The Cu^II^ atom is coordinated by the tridentate *N*-salicylideneglycinato dianion and the 2-methylpyridine ligand in an approximately square-planar geometry. The pyridine ring is inclined with respect to the mean plane (through O1, O2, N1 and N2) at an angle of 11.5 (2) ° and the Cu^II^ atom lies 0.042 (1) Å out of this plane. The Cu—N and Cu—O bond distances [Cu—N 1.919 (2) and 2.018 (2) Å; Cu—O 1.922 (2) and 1.966 (2) Å] do not differ significantly when compared to the 2-ethylpyridine compound (Warda, 1998*c*). These units are associated into dimers through Cu···O interactions (2.680 (2) Å), and these dimers are associated into stacks along the *a* axis of the monoclinic unit cell by longer Cu···O contacts (3.332 (2) Å) (Fig. 2). The Na^+^ cations and perchlorate anions lie between stacks, both on 2-fold axes, forming Na···O contacts of 3.698 (2) Å. The Na^+^ cation is also coordinated by two water molecules in a bent geometry.

### Experimental

The title compound was synthesized from aqua(*N*-salicylideneglycinato)copper(II) hemihydrate according to the methods of Ueki *et al.* (1967) and Warda (1994), with 3-methylpyridine and sodium perchlorate in a 4:3 ethanol-water mixture at room temperature. Dark-green prismatic crystals grew within a few days.

### Refinement

H atoms bonded to C atoms were placed in geometrically idealized positions and constrained to ride on their parent atoms, with C—H = 0.93–0.97 Å and *U*_iso_(H) = 1.2 or 1.5*U*_eq_(C). The H atoms of the water molecules were placed in approximate positions and allowed to ride with O—H = 0.85 Å, *U*_iso_(H) = 1.2 *U*_eq_(O).

### Crystal data

**Table:**

[Na(H_2_O)_2_]ClO_4_·[Cu_2_(C_9_H_7_NO_3_)_2_(C_6_H_7_N)_2_]	*F*_000_ = 844
*M**_r_* = 826.12	*D*_x_ = 1.515 Mg m^−^^3^
Monoclinic, *P*2/*c*	Mo *K*α radiation λ = 0.71073 Å
Hall symbol: -P 2yc	Cell parameters from 7803 reflections
*a* = 7.4677 (5) Å	θ = 2.2–26.9º
*b* = 13.2402 (9) Å	µ = 1.32 mm^−^^1^
*c* = 19.3937 (12) Å	*T* = 292 (2) K
β = 109.165 (2)º	Block, dark-green
*V* = 1811.3 (2) Å^3^	0.30 × 0.26 × 0.24 mm
*Z* = 2	

### Data collection

**Table:**

Bruker SMART APEX CCD diffractometer	3558 independent reflections
Radiation source: sealed tube	2926 reflections with *I* > 2σ(*I*)
Monochromator: graphite	*R*_int_ = 0.047
*T* = 292(2) K	θ_max_ = 26.0º
φ and ω scans	θ_min_ = 1.5º
Absorption correction: multi-scan(SADABS; Bruker, 2000)	*h* = −9→9
*T*_min_ = 0.68, *T*_max_ = 0.73	*k* = −16→16
18486 measured reflections	*l* = −23→23

### Refinement

**Table:**

Refinement on *F*^2^	Secondary atom site location: difference Fourier map
Least-squares matrix: full	Hydrogen site location: inferred from neighbouring sites
*R*[*F*^2^ > 2σ(*F*^2^)] = 0.045	H-atom parameters constrained
*wR*(*F*^2^) = 0.100	*w* = 1/[σ^2^(*F*_o_^2^) + (0.0394*P*)^2^ + 1.073*P*] where *P* = (*F*_o_^2^ + 2*F*_c_^2^)/3
*S* = 1.13	(Δ/σ)_max_ < 0.001
3558 reflections	Δρ_max_ = 0.29 e Å^−^^3^
237 parameters	Δρ_min_ = −0.59 e Å^−^^3^
Primary atom site location: structure-invariant direct methods	Extinction correction: none

### Special details

**Table:**

Geometry. All e.s.d.'s (except the e.s.d. in the dihedral angle between two l.s. planes) are estimated using the full covariance matrix. The cell e.s.d.'s are taken into account individually in the estimation of e.s.d.'s in distances, angles and torsion angles; correlations between e.s.d.'s in cell parameters are only used when they are defined by crystal symmetry. An approximate (isotropic) treatment of cell e.s.d.'s is used for estimating e.s.d.'s involving l.s. planes.
Refinement. Refinement of *F*^2^ against ALL reflections. The weighted *R*-factor *wR* and goodness of fit *S* are based on *F*^2^, conventional *R*-factors *R* are based on *F*, with *F* set to zero for negative *F*^2^. The threshold expression of *F*^2^ > σ(*F*^2^) is used only for calculating *R*-factors(gt) *etc*. and is not relevant to the choice of reflections for refinement. *R*-factors based on *F*^2^ are statistically about twice as large as those based on *F*, and *R*- factors based on ALL data will be even larger.

### Fractional atomic coordinates and isotropic or equivalent isotropic displacement parameters (Å^2^)

**Table:**

	*x*	*y*	*z*	*U*_iso_*/*U*_eq_	Occ. (<1)
C1	0.1080 (4)	0.8274 (2)	0.06545 (17)	0.0359 (6)	
C2	0.0762 (5)	0.7877 (3)	0.1253 (2)	0.0514 (9)	
H2	0.0667	0.8309	0.1618	0.062*	
C3	0.0578 (5)	0.6847 (3)	0.1330 (2)	0.0555 (9)	
H3	0.0363	0.6610	0.1748	0.067*	
C4	0.0695 (5)	0.6178 (3)	0.0832 (2)	0.0566 (10)	
H4	0.0577	0.5489	0.0901	0.068*	
C5	0.1000 (5)	0.6543 (2)	0.0206 (2)	0.0526 (9)	
H5	0.1067	0.6090	−0.0151	0.063*	
C6	0.1203 (4)	0.7558 (2)	0.01060 (18)	0.0411 (7)	
C7	0.1447 (4)	0.7882 (2)	−0.05710 (19)	0.0412 (7)	
H7	0.1370	0.7392	−0.0923	0.049*	
C8	0.1947 (5)	0.8996 (3)	−0.14394 (17)	0.0499 (9)	
H8A	0.0710	0.8977	−0.1814	0.060*	
H8B	0.2744	0.8488	−0.1553	0.060*	
C9	0.2828 (5)	1.0035 (2)	−0.1416 (2)	0.0478 (8)	
C10	0.2917 (4)	1.1111 (3)	0.12608 (17)	0.0427 (7)	
H10	0.2641	1.0502	0.1443	0.051*	
C11	0.3191 (6)	1.1968 (3)	0.1690 (2)	0.0567 (10)	
H11	0.3069	1.1936	0.2152	0.068*	
C12	0.3636 (6)	1.2851 (3)	0.1439 (2)	0.0610 (10)	
H12	0.3780	1.3432	0.1721	0.073*	
C13	0.3882 (5)	1.2894 (3)	0.0754 (2)	0.0547 (9)	
C14	0.3530 (4)	1.1998 (2)	0.03434 (18)	0.0421 (7)	
H14	0.3645	1.2004	−0.0120	0.050*	
C15	0.4383 (6)	1.3829 (3)	0.0458 (2)	0.0591 (10)	
H15A	0.4325	1.4385	0.0768	0.089*	
H15B	0.3508	1.3942	−0.0024	0.089*	
H15C	0.5645	1.3773	0.0436	0.089*	
Cl1	0.0000	0.39685 (9)	0.2500	0.0541 (3)	
Cu1	0.22475 (5)	0.99364 (3)	−0.007850 (19)	0.03341 (12)	
N1	0.1759 (4)	0.87901 (19)	−0.07187 (12)	0.0360 (6)	
N2	0.3039 (3)	1.11394 (19)	0.05920 (13)	0.0351 (5)	
Na1	0.5000	0.60089 (17)	0.2500	0.0603 (5)	
O1	0.1223 (3)	0.92455 (16)	0.05801 (12)	0.0424 (5)	
O2	0.3196 (4)	1.05246 (17)	−0.08228 (13)	0.0500 (6)	
O3	0.3162 (4)	1.0293 (2)	−0.19675 (15)	0.0616 (7)	
O4	0.0491 (4)	0.45860 (19)	0.19803 (14)	0.0574 (7)	
O5	0.1612 (3)	0.33292 (18)	0.28823 (14)	0.0514 (6)	
O6	0.6028 (7)	0.6382 (4)	0.1670 (3)	0.0507 (13)	0.50
H6B	0.5176	0.6692	0.1335	0.061*	0.50
H6C	0.6993	0.6761	0.1836	0.061*	0.50
O7	0.3968 (8)	0.6975 (5)	0.3137 (3)	0.0594 (15)	0.50
H7A	0.3624	0.6626	0.3439	0.071*	0.50
H7B	0.4843	0.7379	0.3367	0.071*	0.50

### Atomic displacement parameters (Å^2^)

**Table:**

	*U*^11^	*U*^22^	*U*^33^	*U*^12^	*U*^13^	*U*^23^
C1	0.0305 (15)	0.0368 (16)	0.0413 (16)	−0.0021 (12)	0.0130 (13)	0.0045 (13)
C2	0.0444 (19)	0.058 (2)	0.048 (2)	−0.0021 (16)	0.0099 (16)	0.0147 (17)
C3	0.052 (2)	0.051 (2)	0.061 (2)	0.0009 (17)	0.0157 (18)	0.0150 (19)
C4	0.055 (2)	0.045 (2)	0.065 (2)	−0.0103 (17)	0.0127 (18)	0.0103 (18)
C5	0.056 (2)	0.0319 (17)	0.066 (2)	−0.0034 (15)	0.0139 (18)	0.0067 (16)
C6	0.0376 (16)	0.0337 (15)	0.0485 (18)	0.0022 (13)	0.0095 (14)	0.0042 (14)
C7	0.0358 (16)	0.0351 (16)	0.0522 (19)	−0.0062 (13)	0.0136 (14)	−0.0014 (14)
C8	0.055 (2)	0.067 (2)	0.0334 (16)	−0.0200 (17)	0.0218 (15)	−0.0045 (16)
C9	0.065 (2)	0.0381 (17)	0.052 (2)	0.0002 (16)	0.0349 (17)	0.0000 (16)
C10	0.0340 (16)	0.053 (2)	0.0415 (17)	−0.0102 (14)	0.0130 (13)	−0.0108 (15)
C11	0.077 (3)	0.056 (2)	0.0450 (19)	−0.0183 (19)	0.0306 (19)	−0.0130 (17)
C12	0.061 (2)	0.058 (2)	0.064 (2)	−0.0098 (19)	0.020 (2)	−0.0130 (19)
C13	0.0367 (18)	0.051 (2)	0.070 (2)	−0.0031 (15)	0.0088 (17)	−0.0139 (18)
C14	0.0388 (17)	0.0453 (18)	0.0434 (18)	−0.0032 (14)	0.0153 (14)	−0.0029 (14)
C15	0.062 (2)	0.048 (2)	0.063 (2)	−0.0155 (18)	0.0148 (19)	−0.0123 (18)
Cl1	0.0648 (8)	0.0430 (6)	0.0597 (7)	0.000	0.0273 (6)	0.000
Cu1	0.0428 (2)	0.02850 (19)	0.0342 (2)	−0.00470 (15)	0.01980 (15)	−0.00310 (14)
N1	0.0465 (15)	0.0336 (13)	0.0289 (12)	−0.0045 (11)	0.0139 (11)	−0.0045 (10)
N2	0.0326 (13)	0.0361 (13)	0.0334 (12)	−0.0041 (10)	0.0066 (10)	−0.0057 (10)
Na1	0.0548 (12)	0.0636 (13)	0.0564 (12)	0.000	0.0101 (10)	0.000
O1	0.0638 (14)	0.0314 (11)	0.0413 (12)	−0.0030 (10)	0.0297 (11)	0.0032 (9)
O2	0.0768 (17)	0.0352 (12)	0.0503 (14)	−0.0093 (11)	0.0377 (13)	−0.0029 (11)
O3	0.0747 (18)	0.0602 (16)	0.0606 (16)	−0.0068 (13)	0.0369 (14)	0.0063 (13)
O4	0.0772 (18)	0.0495 (14)	0.0599 (15)	−0.0009 (13)	0.0419 (14)	−0.0033 (12)
O5	0.0493 (13)	0.0464 (14)	0.0601 (15)	0.0014 (11)	0.0202 (12)	0.0037 (11)
O6	0.036 (3)	0.060 (3)	0.047 (3)	−0.012 (3)	0.001 (2)	−0.012 (3)
O7	0.046 (3)	0.059 (4)	0.065 (4)	−0.009 (3)	0.007 (3)	−0.014 (3)

### Geometric parameters (Å, °)

**Table:**

C1—O1	1.303 (4)	C12—C13	1.401 (6)
C1—C2	1.364 (5)	C12—H12	0.930
C1—C6	1.450 (5)	C13—C14	1.405 (5)
C2—C3	1.384 (5)	C13—C15	1.464 (5)
C2—H2	0.930	C14—N2	1.333 (4)
C3—C4	1.334 (5)	C14—H14	0.930
C3—H3	0.930	C15—H15A	0.960
C4—C5	1.393 (5)	C15—H15B	0.960
C4—H4	0.930	C15—H15C	0.960
C5—C6	1.373 (5)	Cl1—O4	1.436 (3)
C5—H5	0.930	Cl1—O4^i^	1.436 (3)
C6—C7	1.448 (5)	Cl1—O5^i^	1.459 (2)
C7—N1	1.275 (4)	Cl1—O5	1.459 (2)
C7—H7	0.930	Cu1—N1	1.919 (2)
C8—N1	1.475 (4)	Cu1—O1	1.922 (2)
C8—C9	1.519 (5)	Cu1—O2	1.966 (2)
C8—H8A	0.970	Cu1—N2	2.018 (2)
C8—H8B	0.970	Na1—O6	2.058 (6)
C9—O3	1.224 (4)	Na1—O6^ii^	2.058 (6)
C9—O2	1.269 (4)	Na1—O7^ii^	2.093 (6)
C10—N2	1.330 (4)	Na1—O7	2.093 (6)
C10—C11	1.382 (5)	O6—H6B	0.850
C10—H10	0.930	O6—H6C	0.850
C11—C12	1.349 (5)	O7—H7A	0.850
C11—H11	0.930	O7—H7B	0.850
			
O1—C1—C2	121.2 (3)	C14—C13—C15	121.1 (4)
O1—C1—C6	122.5 (3)	N2—C14—C13	122.5 (3)
C2—C1—C6	116.3 (3)	N2—C14—H14	118.7
C1—C2—C3	121.5 (4)	C13—C14—H14	118.7
C1—C2—H2	119.2	C13—C15—H15A	109.5
C3—C2—H2	119.2	C13—C15—H15B	109.5
C4—C3—C2	122.9 (4)	H15A—C15—H15B	109.5
C4—C3—H3	118.5	C13—C15—H15C	109.5
C2—C3—H3	118.5	H15A—C15—H15C	109.5
C3—C4—C5	118.0 (3)	H15B—C15—H15C	109.5
C3—C4—H4	121.0	O4—Cl1—O4^i^	110.6 (2)
C5—C4—H4	121.0	O4—Cl1—O5^i^	109.39 (15)
C6—C5—C4	121.3 (4)	O4^i^—Cl1—O5^i^	109.18 (14)
C6—C5—H5	119.3	O4—Cl1—O5	109.18 (14)
C4—C5—H5	119.3	O4^i^—Cl1—O5	109.39 (15)
C5—C6—C7	118.1 (3)	O5^i^—Cl1—O5	109.1 (2)
C5—C6—C1	120.0 (3)	N1—Cu1—O1	91.20 (10)
C7—C6—C1	121.9 (3)	N1—Cu1—O2	82.87 (10)
N1—C7—C6	124.7 (3)	O1—Cu1—O2	174.01 (9)
N1—C7—H7	117.6	N1—Cu1—N2	174.05 (10)
C6—C7—H7	117.6	O1—Cu1—N2	92.51 (10)
N1—C8—C9	108.3 (3)	O2—Cu1—N2	93.47 (10)
N1—C8—H8A	110.0	C7—N1—C8	117.9 (3)
C9—C8—H8A	110.0	C7—N1—Cu1	127.6 (2)
N1—C8—H8B	110.0	C8—N1—Cu1	114.2 (2)
C9—C8—H8B	110.0	C10—N2—C14	119.5 (3)
H8A—C8—H8B	108.4	C10—N2—Cu1	120.4 (2)
O3—C9—O2	127.1 (3)	C14—N2—Cu1	119.8 (2)
O3—C9—C8	115.9 (3)	O6—Na1—O6^ii^	152.2 (3)
O2—C9—C8	116.9 (3)	O6^ii^—Na1—O7^ii^	128.2 (3)
N2—C10—C11	121.3 (3)	O6—Na1—O7	128.2 (3)
N2—C10—H10	119.3	O7^ii^—Na1—O7	104.7 (4)
C11—C10—H10	119.3	C1—O1—Cu1	127.5 (2)
C12—C11—C10	120.0 (3)	C9—O2—Cu1	116.1 (2)
C12—C11—H11	120.0	Na1—O6—H6B	109.6
C10—C11—H11	120.0	Na1—O6—H6C	109.5
C11—C12—C13	120.1 (4)	H6B—O6—H6C	109.4
C11—C12—H12	119.9	Na1—O7—H7A	109.1
C13—C12—H12	119.9	Na1—O7—H7B	109.0
C12—C13—C14	116.4 (4)	H7A—O7—H7B	109.6
C12—C13—C15	122.4 (3)		

Symmetry codes: (i) −*x*, *y*, −*z*+1/2; (ii) −*x*+1, *y*, −*z*+1/2.
